# Lemur tyrosine kinase 2 silencing inhibits the proliferation of gastric cancer cells by regulating GSK-3β phosphorylation and β-catenin nuclear translocation

**DOI:** 10.1080/21655979.2021.1999375

**Published:** 2022-03-01

**Authors:** Xin Han, Da-Zhong Wang, Meng Yuan, Wei-Jun Bai

**Affiliations:** Department of Thoracic Cancer, Cancer Hospital of China Medical University, Liaoning Cancer Hospital and Institute, Shenyang, Liaoning, P.R. China

**Keywords:** Gastric cancer, lemur tyrosine kinase 2, proliferation, GSK-3β, β-catenin

## Abstract

Previous studies on the mechanism of proliferation and cell cycle progression of gastric cancer cells have shown promising perspectives for the prevention and treatment of gastric cancer. The aim of the present study was to investigate the role of lemur tyrosine kinase 2 (LMTK2) in gastric cancer cell proliferation and cell cycle progression, as well as in tumor-bearing nude mouse models. The expression levels of LMTK2 were determined in gastric cancer cell lines. In addition, the effects of LMTK2 silencing or overexpression on cell proliferation were measured using Cell Counting Kit-8, BrdU and colony formation assays. Cell cycle progression was analyzed using flow cytometry and western blotting. The expression levels of proteins associated with the β-catenin pathway were assessed using western blot analysis. A tumor-bearing nude mouse model was established by injecting gastric cancer cells, and the effect of LMTK2 knockdown or overexpression on tumor growth was examined. The expression levels of LMTK2 were found to be upregulated in all gastric cancer cell lines. Moreover, LMTK2 knockdown inhibited cell proliferation, colony formation and cell cycle progression. LMTK2 knockdown also inhibited the activation of GSK-3β/β-catenin signaling, as evidenced by reduced GSK-3β phosphorylation and nuclear β-catenin levels. LMTK2 knockdown also suppressed tumor growth, whereas overexpression accelerated this process. In conclusion, LMTK2 silencing can inhibit the proliferation of gastric cancer cells *in vitro* and tumor growth *in vivo* by regulating GSK-3β phosphorylation and β-catenin nuclear translocation.

## Introduction

Gastric cancer is the fifth most common cancer and the third leading cause of cancer-associated death worldwide [1]. Risk factors for the occurrence of gastric cancer include *Helicobacter pylori* infection, age and diet [2,3]. Gastric cancer is a disease with high molecular and phenotypic heterogeneity [4]. Due to a lack of obvious symptoms in its early stages, this condition can easily be mistaken for indigestion [5]. Gastric cancer is diagnosed using endoscopic biopsy and histology, followed by imaging examination and endoscopic ultrasound for staging [6]. The main treatment option for early gastric cancer is endoscopic resection [7]. For later stage operable gastric cancer, surgery, including D2 lymph node dissection, is used to remove the cancerous tissue. Moreover, perioperative or adjuvant chemotherapy can be essential in order to improve the survival rate of the patients [8]. Patients who are diagnosed with advanced gastric cancer require continuous chemotherapy, usually with first-line dual-drug fluorouracil and cisplatin treatment [9]. Currently, there are several targeted drugs approved for the treatment of gastric cancer, including the first-line trastuzumab for HER2^+^ patients, the second-line VEGFR2 antagonist remucirumab and the third-line programmed cell death 1 (PD1) inhibitors nivolumab and pembrolizumab. However, the long-term survival rate remains poor [1].

Gastric cancer is the result of accumulative damage to the genome, which affects cell function and is considered a prerequisite of tumor progression [10]. Lemur tyrosine kinase 2 (LMTK2) belongs to the serine/threonine protein kinase family of transmembrane proteins [11]. LMTK2 disorders are involved in a variety of pathological processes, including cancer, infertility, cystic fibrosis and neurodegeneration. Zhang *et al* [12] demonstrated that the LMTK2 protein was highly expressed in colon cancer cells and that its silencing inhibited proliferation. In addition, Conti *et al* [13] suggested that LMTK2 could determine breast and colorectal cancer cell sensitivity to apoptosis by regulating the levels of BCL2 family members. Zhao *et al* [14] reported that the expression levels of LMTK2 in hepatocellular carcinoma tissue were upregulated and that high expression of LMTK2 was associated with reduced survival times. However, the specific role of LMTK2 in gastric cancer remains unclear.

Previous studies on the mechanism of proliferation and cell cycle progression of gastric cancer cells have shown promising perspectives for the prevention and treatment of gastric cancer [15]. Further research in this direction may help further elucidate the pathogenesis of gastric cancer, whilst identifying novel targets for the treatment of this disease. Understanding the pathways, the oncogenes and the tumor suppressor genes involved may provide the means to uncover novel therapeutic targets or prognostic markers of therapeutic responsiveness [16].

Therefore, the aim of the present study was to investigate the role of LMTK2 in gastric cancer cell proliferation and cell cycle progression, as well as in tumor-bearing nude mouse models.

## Materials and methods

### Cell lines and transfection

The GEL-1 normal gastric epithelial cells and the KATO III, HSC-39, AGS and MKN45 gastric cancer cell lines were obtained from the American Type Culture Collection. Cells were cultured in DMEM (Gibco; Thermo Fisher Scientific, Inc.) supplemented with 10% FBS (Gibco; Thermo Fisher Scientific, Inc.) in an incubator set at 37°C and 5% CO_2_.

Two types of short hairpin RNA (shRNA) targeting LMTK2, as well as a scrambled shRNA used as a negative control (NC), were inserted into the pLKO.1 plasmid [17]. For overexpression, the pcDNA3.1 vector containing LMTK2 (Ov-LMTK2) was used, together with the empty vector as NC. The shRNA molecules and plasmids were obtained from Shanghai GenePharma Co., Ltd. The shRNA (50 nM) or pcDNA3.1 (20 µg) were transfected into AGS and MKN45 cells (5x10^5^ cells/well) using Lipofectamine® 2000 (Invitrogen; Thermo Fisher Scientific, Inc.) according to the manufacturer’s instructions. Following transfection for 6 h, the medium was replaced with normal culture medium and cells were incubated for another 48 h. At the end of this incubation, the expression levels of LMTK2 were determined.

### Animals and preparation of tumor transplants

All procedures followed the ethical guidelines of Cancer Hospital of China Medical University. All efforts were made to minimize animal suffering. The experimental procedures were approved by The Research and Education Animal Use and Care Committee of Cancer Hospital of China Medical University. The experiments [18] were carried out using 30 adult male Balb/c nude mice (age, 4–6 weeks; weight, 16–18 g; Jiangsu AI LING FEI Co., Ltd.), housed at a constant ambient temperature of 21 ± 2°C and a relative humidity of 55 ± 5%, with a 12-h light/dark cycle, and free access to food and water. For each group (AGS, shRNA-LMTK2 and Ov-LMTK2), 1 × 10^6^ cells were separately resuspended in a 0.2-ml volume. The right part of the abdomen was wiped with alcohol using a cotton ball, and the cell suspension was then injected using a syringe. After the needle was pulled out, the injection site was dried with a sterile cotton swab. The size of the tumors and the weight of the mice were observed every 2 days, and photographs were obtained. When the humane endpoints were reached (for example, difficulty to eat), the experiment was terminated, and the mice were euthanized using an overdose of sodium pentobarbital (100 mg/kg by intraperitoneal injection) followed by cervical dislocation. A total of 2 weeks later, after the nude mice were sacrificed in the same way, the tumor tissue was taken out for the subsequent assays.

### Western blotting

Protein was extracted from tissue samples or cells using RIPA lysis buffer, and the protein concentration was determined using a Bradford assay. Protein (25 μg) was separated on a 6 or 10% polyacrylamide gel, then transferred to PVDF membranes. The membranes were blocked with TBS Tween-20 buffer containing 5% BSA at room temperature for 1 h, then incubated with the following primary antibodies overnight at 4°C: LMTK2 (cat. no. ab172622; 1:1,000), CDK4 (cat. no. ab108357; 1:1,000), CDK6 (cat. no. ab124821; 1:1,000), cyclin D1 (cat. no. ab16663; 1:200), GSK-3β (cat. no. ab93926; 1:500), phosphorylated (p)-GSK-3β (cat. no. ab75814; 1:10,000), β-catenin (cat. no. ab32572; 1:10,000), lamin B1 (cat. no. ab16048; 1;10,000), Ki67 (cat. no. ab16667; 1:1,000) and proliferating cell nuclear antigen (PCNA; cat. no. ab29; 1:1,000). The membranes were then incubated with the HRP-conjugated goat anti-rabbit (cat. no. ab97051; 1:20,000) or rabbit anti-mouse secondary antibody (cat. no. ab6728; 1:2,000) for 1 h at room temperature. All antibodies were obtained from Abcam. Protein bands were visualized using electrochemiluminescence detection reagent (MilliporeSigma). The results were analyzed using ImageJ version 1.52 software (National Institutes of Health) [19].

### Reverse transcription-quantitative PCR (RT-qPCR)

Total RNA was extracted from cultured cells using TRIzol® reagent (Invitrogen; Thermo Fisher Scientific, Inc.). RNA samples were reverse transcribed into cDNA using PrimeScript™ RT Master Mix (Takara Biotechnology Co., Ltd.). qPCR was carried out using the QuantiTect SYBR-Green PCR kit (Qiagen, Inc.). The qPCR conditions consisted of a single cycle at 95°C for 20 sec, followed by 50 cycles at 95°C for 1 sec and 60°C for 20 sec. The relative expression levels were quantified using the 2^−∆∆Cq^ method [20] following normalization against GAPDH. The sequences of the primers used were as follows: LMTK2 forward, 5ʹ-CCTTGTCATTCTCCCACCCC-3ʹ and reverse, 5ʹ- CTCGGAGGAGAAGATTCGGC-3ʹ; and GAPDH forward, 5ʹ- GACTCATGACCACAGTCCATGC-3ʹ and reverse, 5ʹ-AGAGGCAGGGATGATGTTCTG-3ʹ.

### Cell Counting Kit-8 (CCK-8) assay

To assess the effect of LMTK2 expression on cell proliferation, untransfected and transfected AGS and MKN45 cells (5x10^3^ cells/well) were seeded into the wells of a 96-well plate. Cells were then incubated at 37°C for 24, 48 and 72 h. For each time point, the cells were incubated in 10 µl CCK-8 solution (Dojindo Molecular Technologies, Inc.) at 37°C for 3 h. Absorbance was then measured at 450 nm using a microplate reader (Bio-Rad Laboratories, Inc.) [21].

### 5-bromo-2ʹ-deoxyuridine (BrdU) staining

AGS and MKN45 cells (5x10^3^ cells/well) were seeded into 96-well plate and cultured until 50–60% confluence. BrdU (10 μl/well; 10 μM) was then added, and the cells were incubated at 37°C for 48 h. After washing three times with PBS, the cells were fixed with 4% paraformaldehyde for 30 min at 4°C. After washing three times with PBS, cells were incubated with 0.5 ml 2 M HCl for 5 min at 37°C. Sodium borate (0.1 M) was used for neutralization. A volume of 1 ml 0.2% TritonX-100 was then added for 10 min. The cells were blocked with 3% BSA for 1 h at room temperature, then incubated with anti-BrdU antibody (cat. no. ab8152; 1:200; Abcam) at 4°C overnight. The samples were then incubated with FITC-conjugated goat anti-mouse antibody (cat. no. ab6785; 1:1,000; Abcam) for 1 h at room temperature. The stained cells were observed under a fluorescence microscope (magnification, x100; Olympus Corporation) [22].

### Colony formation assay

AGS and MKN45 cells were uniformly seeded into culture dishes at a density of 500 cells/dish, then cultured at 37°C with 5% CO_2_ and saturated humidity for 2 weeks. The medium was changed every 3 days. After 2 weeks, the supernatant was discarded, and the dishes were washed carefully with PBS twice. The cells were fixed with 4% paraformaldehyde for 15 min at room temperature. The fixative solution was then replaced with Crystal violet to stain the cells for 30 min at room temperature. A cluster of >50 cells was considered as a colony [23].

### Flow cytometry

AGS and MKN45 cells were lysed and washed twice with PBS. The cells were then permeabilized with 50 μg/ml Triton-X100 (Sigma-Aldrich; Merck KGaA) and stained with 0.15% propidium iodide (Thermo Fisher Scientific, Inc.) in the dark at 4°C for 15 min. The data were acquired using a FACSCalibur flow cytometer (Becton, Dickinson and Company), and the results were analyzed using FlowJo software (version 7.6.1; FlowJo LLC) [24].

### Bioinformatics and statistical analysis

The expression levels of LMTK2 in patients with different types of cancer were obtained from the Broad Institute Cancer Cell Line Encyclopedia (CCLE) website (portals.broadinstitute.org/ccle) [25]. All experiments were conducted independently three times. Statistical analysis was performed using Prism 8.0 software (GraphPad Software, Inc.). The data are presented as the mean ± standard deviation. A one-way ANOVA followed by a Tukey’s post hoc test was used to compare the differences between multiple groups. P < 0.05 was considered to indicate a statistically significant difference.

## Results

The present study was to investigate the role of LMTK2 in gastric cancer cell proliferation and cell cycle progression, as well as in tumor-bearing nude mouse models. The expression levels of LMTK2 were found to be upregulated in gastric cancer cell lines. Moreover, LMTK2 knockdown inhibited cell proliferation, colony formation and cell cycle progression. LMTK2 knockdown also inhibited the activation of GSK-3β/β-catenin signaling, as evidenced by reduced GSK-3β phosphorylation and nuclear β-catenin levels. LMTK2 knockdown also suppressed tumor growth, whereas overexpression accelerated this process. Briefly, LMTK2 silencing can inhibit the proliferation of gastric cancer cells *in vitro* and tumor growth *in vivo* by regulating GSK-3β phosphorylation and β-catenin nuclear translocation.

### Expression levels of LMTK2 in gastric cancer

**Figure 1.**
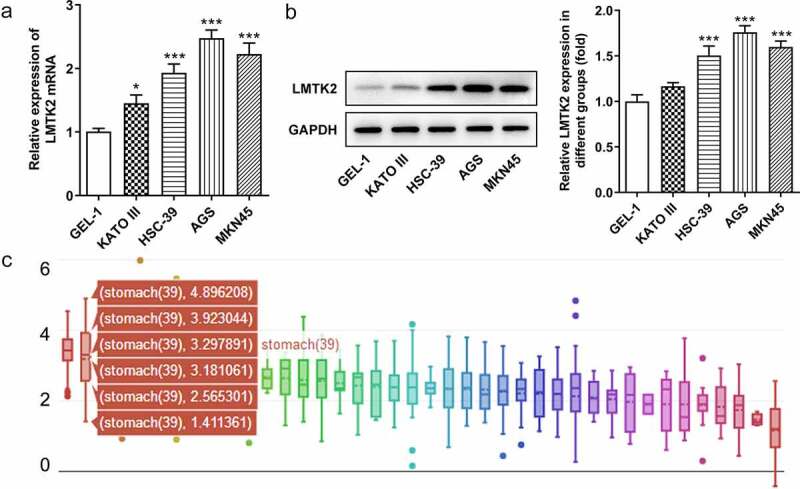
The expression levels of LMTK2 in the GEL-1 normal gastric epithelial cell line and gastric cancer cell lines (KATO III, HSC-39, AGS and MKN45) were determined using (a) reverse transcription-quantitative PCR and (b) western blotting. (c) The expression levels of LMTK2 in several types of cancer were obtained from the Cancer Cell Line Encyclopedia website. ***P < 0.001, *P < 0.05.

The expression levels of LMTK2 were examined in the GEL-1 normal gastric epithelial cell line and four gastric cancer cell lines (KATO III, HSC-39, AGS and MKN45) using RT-qPCR and western blotting. The expression levels of LMTK2 were upregulated in all gastric cancer cell lines compared with the GEL-1 normal epithelial cells. As the expression levels were highest in AGS and MKN45 cells, these cell lines were used for subsequent experiments (Figure 1(a) and (b)). In addition, the expression levels of LMTK2 in patients with different types of cancer were obtained from the CCLE website. The expression levels of LMTK2 were upregulated in patients with gastric cancer (Figure 1(c)).

### LMTK2 silencing inhibits cell proliferation, colony formation and cell cycle progression

**Figure 2.**
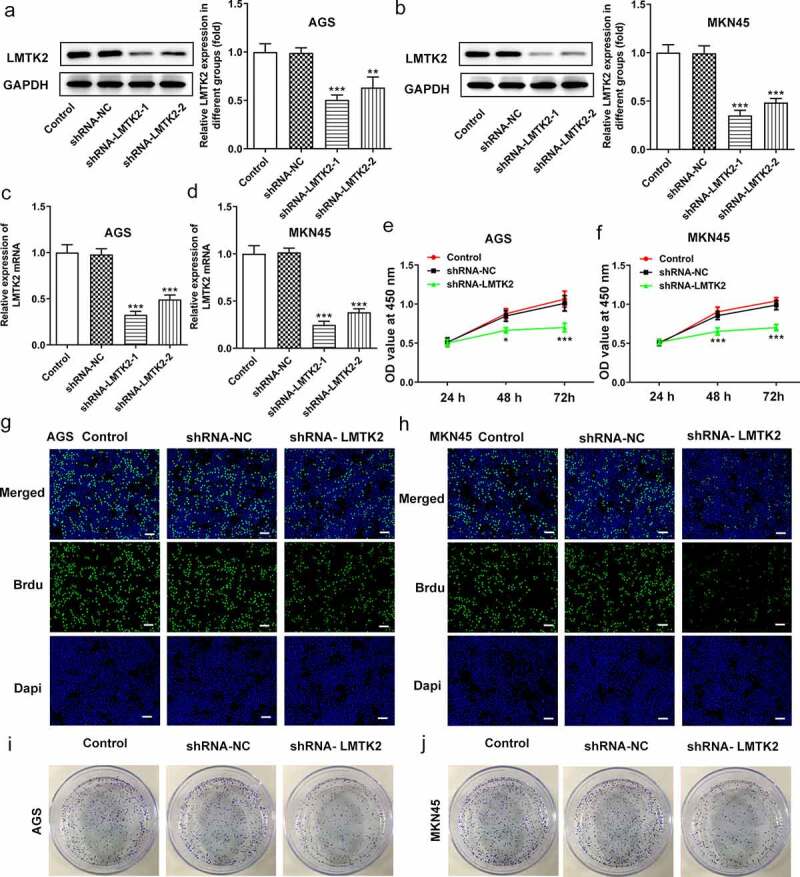
The efficiency of LMTK2 silencing in the AGS cell line was determined using (a) western blotting and (b) RT-qPCR. The efficiency of LMTK2 silencing in the MKN45 cell line was determined using (c) western blotting and (d) RT-qPCR. Cell proliferation was assessed in (e) the AGS and (f) the MKN45 cell lines using Cell Counting Kit-8 assays. Cell proliferation was assessed in (g) the AGS and (h) the MKN45 cell lines using BrdU staining. Colony formation assays were carried out in (i) the AGS and (j) the MKN45 cell lines. ***P < 0.001, *P < 0.05.

The efficiency of shRNA transfection was determined using RT-qPCR and western blotting. AGS and MKN45 cells were divided into four groups: Control, shRNA-NC, shRNA-LMTK2-1 and shRNA-LMTK2-2. For both cell lines, the expression levels of LMTK2 were downregulated in the shRNA-LMTK2-1 group (Figure 2(a-d)). This shRNA group was used in subsequent experiments.

Cell proliferation was assessed using CCK-8 assays, BrdU staining and colony formation assays. The CCK-8 assays demonstrated that the proliferation of the shRNA-LMTK2-transfected cells was lower than that of the control and shRNA-NC groups at all time points (24, 48 or 72 h; Figure 2(e) and (f)). In the BrdU assays, the fluorescence intensity of the shRNA-LMTK2 group in both cell lines was significantly downregulated (Figure 2(g) and (h)). In addition, the number of colonies in the shRNA-LMTK-transfected group was also reduced (Figure 2(i) and (j)).

**Figure 3.**
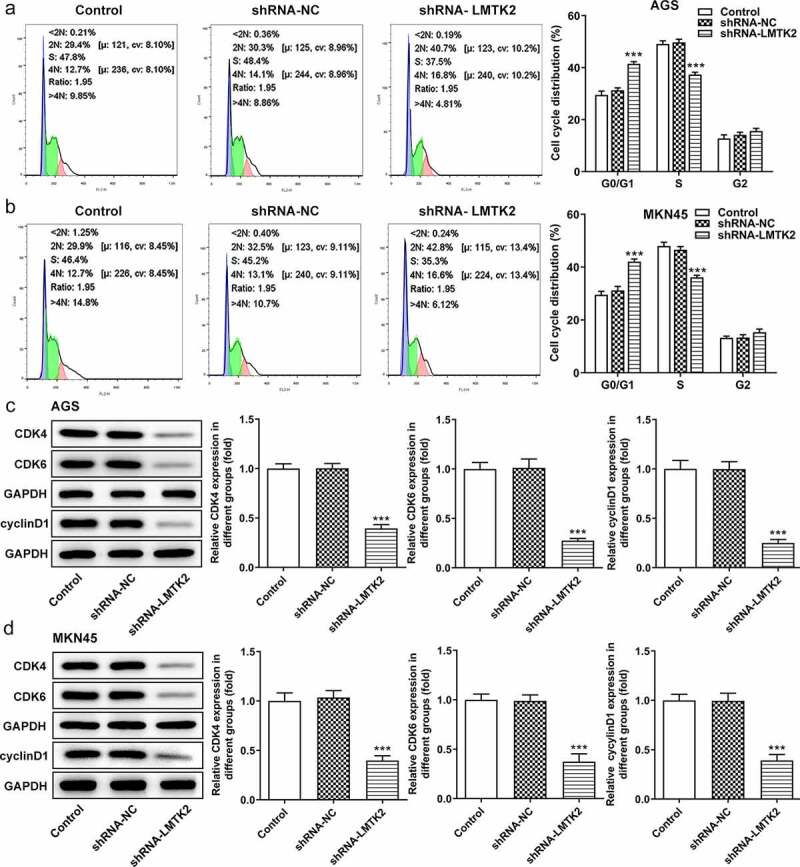
Cell cycle progression was analyzed in (a) the AGS and (b) the MKN45 cell lines using flow cytometry. The expression levels of CDK4, CDK6 and cyclin D1 in (c) the AGS and (d) the MKN45 cell lines were measured using western blotting. ***P < 0.001.

Furthermore, the distribution of cells in the G_1_, S and G_2_/M phases of the cell cycle was examined using flow cytometry. Compared with the shRNA-NC group, the number of shRNA-LMTK2-transfected AGS cells in the G_1_ phase was increased, which was accompanied by a reduction in the number of cells in the S phase. The same trend was observed in the MKN45 cell line (Figure 3(a) and (b)). Moreover, the expression levels of CDK4, CDK6 and cyclin D1 were measured using western blotting and were found to be downregulated in shRNA-LMTK2-transfected cells, suggesting that cell cycle progression was inhibited (Figure 3(c) and (d)).

### LMTK2 silencing suppresses the GSK-3β/β-catenin signaling pathway

**Figure 4.**
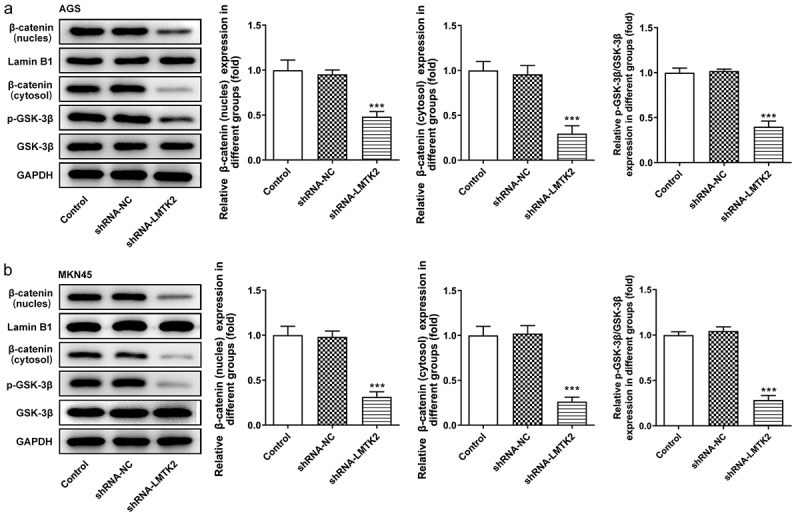
The expression levels of GSK-3β, p-GSK-3β, β-catenin (nucleus) and lamin B1 in (a) the AGS and (b) the MKN45 cell lines were assessed using western blotting. ***P < 0.001.

Based on the aforementioned findings, the activated GSK-3β/β-catenin signaling pathway was studied. The expression levels of GSK-3β, p-GSK-3β, β-catenin (nucleus), β-catenin (cytosol) in AGS and MKN45 cells were assessed using western blotting. The expression levels of p-GSK-3β were significantly downregulated in shRNA-LMTK2-transfected cells, whereas those of GSK-3β remained unchanged, suggesting LMTK2 silencing could inhibit GSK-3β phosphorylation. In addition, the expression levels of β-catenin in the nucleus and cytosol were downregulated. This suggested that LMTK2 knockdown may promote the degradation of β-catenin and inhibit its translocation to the nucleus (Figure 4(a) and (b)).

### LMTK2 promotes cell proliferation, colony formation and cell cycle progression through the GSK-3β/β-catenin signaling pathway

**Figure 5.**
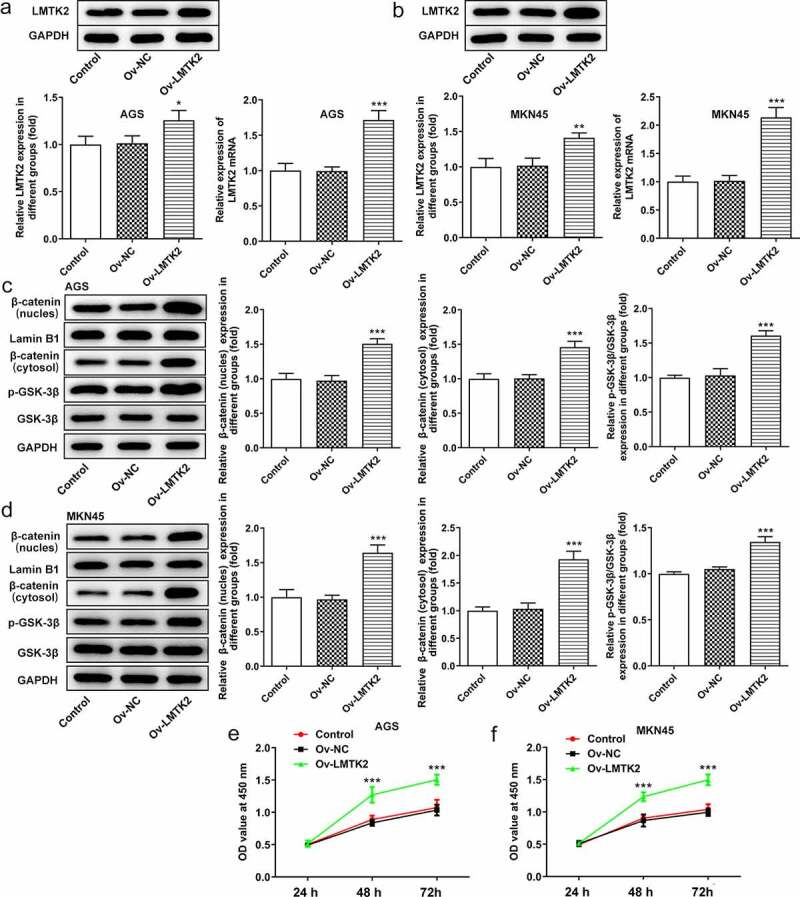
The efficiency of LMTK2 overexpression in (a) the AGS and (b) the MKN45 cell lines was determined using western blotting and RT-qPCR. The expression levels of GSK-3β, p-GSK-3β, β-catenin (nucleus) and lamin B1 in (c) the AGS and (d) the MKN45 cell lines were assessed using western blotting. Cell proliferation was assessed in (e) the AGS and (f) the MKN45 cell lines using Cell Counting Kit-8 assays. Cell proliferation was assessed in (g) the AGS and (h) the MKN45 cell lines using BrdU staining. Colony formation assays were carried out in (i) the AGS and (j) the MKN45 cell lines. ***P < 0.001, *P < 0.05.

The Ov-LMTK2 vector was transfected into AGS and MKN45 cells in order to examine the potential role of the GSK-3β/β-catenin signaling pathway. The efficiency of transfection was determined using RT-qPCR and western blotting. AGS and MKN45 cells were divided into three groups: Control, Ov-NC and Ov-LMTK2. The expression levels of LMTK2 in the Ov-LMTK2 group were significantly upregulated, suggesting successful transfection (Figure 5(a) and (b)).

**Figure 6.**
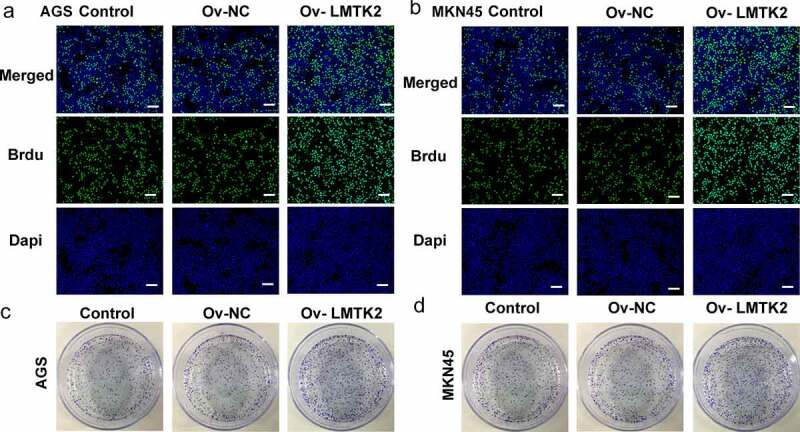
Cell cycle progression was analyzed in (a) the AGS and (b) the MKN45 cell lines using flow cytometry. The expression levels of CDK4, CDK6 and cyclin D1 in (c) the AGS and (d) the MKN45 cell lines were measured using western blotting. ***P < 0.001, **P < 0.01, *P < 0.05.

**Figure 7.**
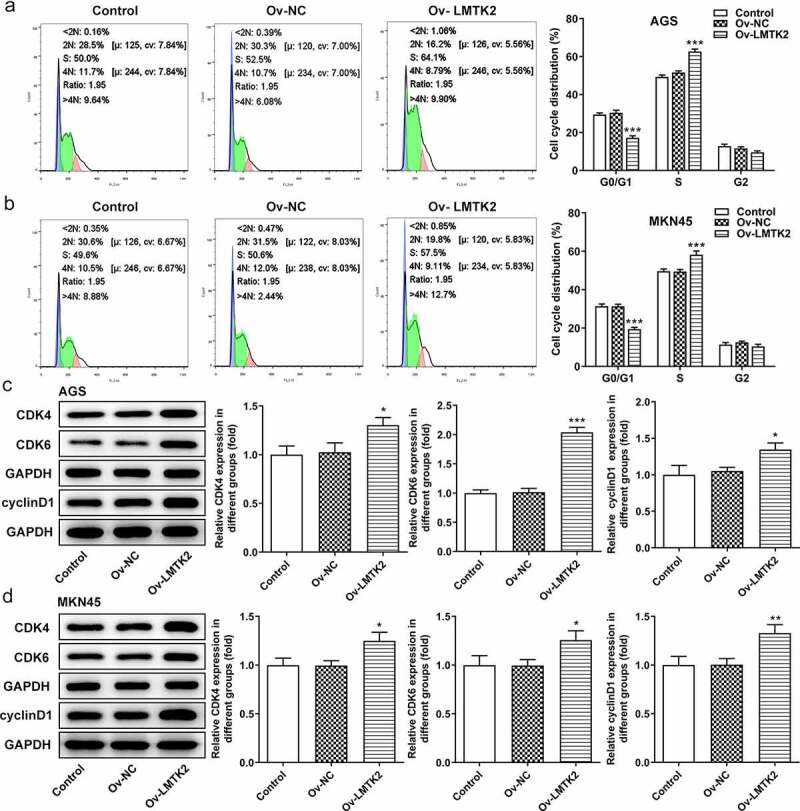
(a) Images of nude mice and tumors. (b) The weights of the mice were recorded every 2 days. (c) The sizes of the tumors were recorded every 2 days. (d) The weights of the tumors were recorded on the last day of the experiment. (e) The expression levels of proteins associated with the cell cycle (CDK4, CDK6 and cyclin D1) or with proliferation (Ki67 and PCNA) were determined in tumor tissue samples using western blotting. (f) The expression levels of GSK-3β, p-GSK-3β, β-catenin (nucleus) and lamin B1 were determined in tumor tissue samples using western blotting. ***P < 0.001, **P < 0.01, *P < 0.05.

The expression levels of GSK-3β, p-GSK-3β, β-catenin (nucleus) and β-catenin (cytosol) were assessed using western blotting. The levels of p-GSK-3β and β-catenin were upregulated following LMTK2 overexpression (Figure 5(c) and (d)). Moreover, the effects of LMTK2 overexpression on cell proliferation were assessed using CCK-8 assays, BrdU staining and colony formation assays. The results of these three assays demonstrated that LMTK2 overexpression promoted cell proliferation (Figure 5(e-f) and 6(a-d)). In addition, the role of LMTK2 overexpression on cell cycle progression was examined using flow cytometry and western blotting Figure 6. LMTK2 overexpression resulted in a reduction in the number of cells in the G_1_ phase and an increase in the number of cells in the S phase (Figure 7(a) and (b)). Similarly, western blot analysis suggested that the protein expression levels of cyclin and cyclin-dependent kinases were upregulated in the Ov-LMTK2 group (Figure 7(c) and (d)).

### *Effect of LMTK2 on* in vivo *gastric tumor growth*

**Figure 8.**
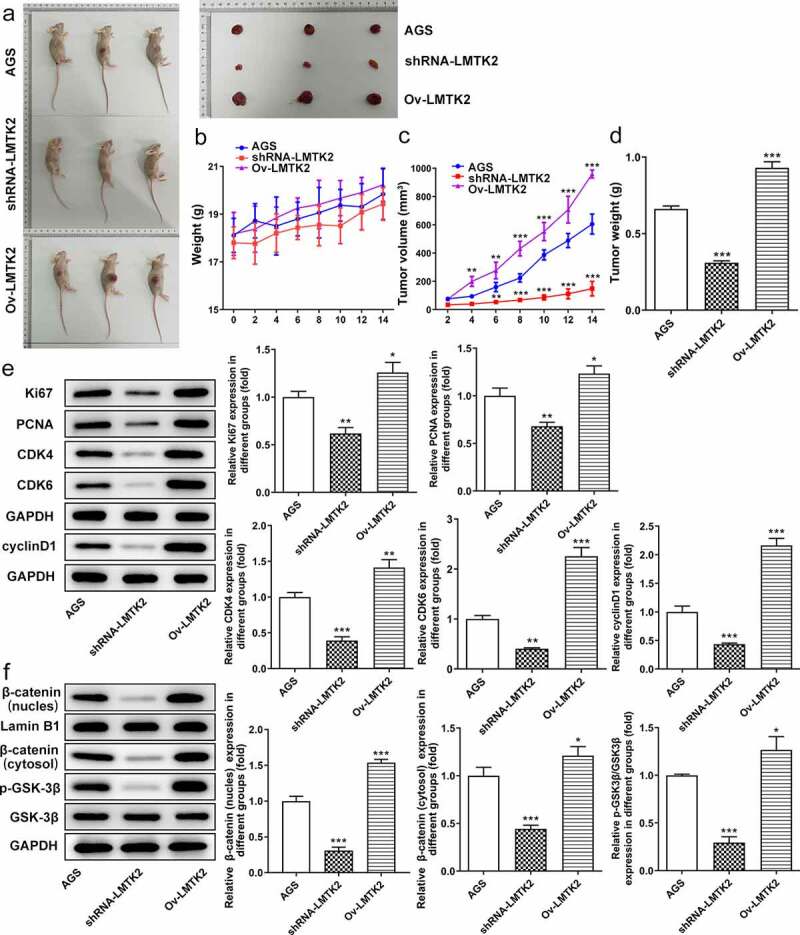
The weights calculation of the mice in the Ov-LMTK2 group.

The sizes of the tumors and the weights of the mice were recorded every 2 day. The weights of the tumors were recorded on the last day of the experiment. The weight of the mice in the shRNA-LMTK2 group increased over time but did not exceed those of the AGS group. The weights of the mice in the Ov-LMTK2 group were greater than those of the AGS group on day 4, then continued to rise (Figure 8(a) and (b)). Moreover, compared with the AGS group, the size of the tumors increased slowly in the shRNA-LMTK2 group, but sharply in the Ov-LMTK2 group (Figure 8(c)). The weights of the tumors were also increased in the Ov-LMTK2 group, but reduced in the shRNA-LMTK2 group (Figure 8(d)). The expression levels of proteins associated with the cell cycle (CDK4, CDK6 and cyclin D1), proliferation (Ki67 and PCNA) and the pathway (GSK-3β, p-GSK-3β, β-catenin) in tumor tissue samples were determined using western blotting. Compared with the AGS group, the expression levels of CDK4, CDK6, cyclin D1, Ki67 and PCNA were downregulated in the shRNA-LMTK2 group and upregulated in the Ov-LMTK2 group (Figure 8(e)). In addition, the expression levels of p-GSK-3β and β-catenin were downregulated in the shRNA-LMTK2 group and upregulated in the Ov-LMTK2 group (Figure 8(f)).

## Discussion

Gastric cancer remains a major threat to human health. Excessive intake of salt/salty foods, as well as insufficient intake of fresh fruits and vegetables, can increase the risk of gastric cancer [26]. It is well-documented that daily eating habits are closely associated with the occurrence of gastric diseases, including gastric cancer [27]. Epidemiological data suggest that prior history of digestive problems, as well as a family history of tumors, represent important risk factors for the onset of gastric cancer [28]. Although surgical resection and chemotherapy are the primary strategies for the treatment of gastric cancer, the survival rate after treatment remains poor [29]. There is a need for improved sanitation, increased intake of fresh fruits and vegetables, safe food preservation methods and smoking reduction, in order to reduce the risk of gastric cancer [30]. The etiology and pathogenesis of gastric cancer, have not yet been fully elucidated, and targeted therapy has always been considered to be a promising option in the treatment of cancer [31]. In the present study, the effect of LMTK2 on the proliferation of gastric cancer cells was examined, and the possible mechanism was explored.

GSK-3β is a serine protein kinase that can regulate the function of substrates through phosphorylation and participates in physiological and pathological processes, such as glycogen metabolism, cell cycle regulation, apoptosis, cell differentiation and inflammation [32]. β-catenin is an important regulatory protein of the Wnt signaling pathway that is regulated by ubiquitination and degradation [33]. GSK-3β can phosphorylate β-catenin, which in turn is degraded in the cytoplasm and loses the ability to enter the nucleus to initiate downstream gene transcription [34]. However, auto-phosphorylation of GSK3 can inhibit its own activity [35,36]. Kim *et al* found that a naturally synthesized carotenoid could downregulate the expression of p-GSK-3β and nuclear β-catenin in AGC gastric cancer cells [37]. Ma *et al* knocked out a tumor suppressor gene and observed that the expression of p-GSK-3β and β-catenin in the SGC-7901 and HGC-27 gastric cancer cell lines was significantly upregulated, which promoted cell migration and invasion [38]. Similarly, in the present study, LMTK2 silencing downregulated the expression levels of p-GSK-3β and nuclear β-catenin *in vitro* (AGS and MKN45 cell lines) and *in vivo*, which was accompanied by stable total-GSK-3β expression.

GSK-3β has also been studied in other cancer types. In breast cancer, the overexpression of a GSK-3β mutant acts as an inhibitor of endogenous wild-type GSK-3β protein, increases the sensitivity of breast cancer to chemotherapeutic compounds and blocks cell cycle progression [39]. In pancreatic cancer, knockdown of GSK-3β leads to a decrease in Bcl-2 and VEGF levels, thereby inhibiting tumor growth and angiogenesis [40]. GSK-3β plays an important role in cancer types that are resistant to chemotherapy, radiotherapy and targeted therapy. Furthermore, Bao *et al* consider that LMTK2 targets GSK-3β and regulates GSK-3β phosphorylation [41]. The present study was limited to studying the effect of LMTK2 silencing on the GSK-3β/β-catenin pathway, cell proliferation and cell cycle progression. Its effect on gastric cancer cell apoptosis and drug resistance should be examined in future studies.

## Conclusion

In conclusion, the expression of LMTK2 in gastric cancer cells was upregulated. Additionally, LMTK2 silencing could suppress cell proliferation, colony formation and cell cycle progression through the GSK-3β/β-catenin signaling pathway. Moreover, LMTK2 silencing could inhibit the growth of gastric tumors *in vivo*. Thus, these findings indicate that LMTK2 may represent a potential target for gastric cancer treatment, which provides a novel direction for future therapeutic strategies.

## Data Availability

The datasets generated and/or analyzed during the current study are available from the corresponding author upon reasonable request.
